# Large-volume paracentesis effects plasma disappearance rate of indo-cyanine green in critically ill patients with decompensated liver cirrhosis and intraabdominal hypertension

**DOI:** 10.1186/s13613-018-0422-6

**Published:** 2018-07-06

**Authors:** Ulrich Mayr, Leonie Fahrenkrog-Petersen, Gonzalo Batres-Baires, Alexander Herner, Sebastian Rasch, Roland M. Schmid, Wolfgang Huber, Tobias Lahmer

**Affiliations:** Klinik und Poliklinik für Innere Medizin II, Klinikum rechts der Isar, Technische Universität München, Ismaninger Strasse 22, 81675 Munich, Germany

**Keywords:** Indo-cyanine green (ICG), Plasma disappearance rate (PDR), Large-volume paracentesis (LVP), Intraabdominal hypertension (IAH), Intraabdominal pressure (IAP), Decompensated liver cirrhosis, Hepatosplanchnic blood flow, Transpulmonary thermodilution, Color-coded duplex sonography, Hepatic artery resistance index

## Abstract

**Background:**

Ascites is a major complication of decompensated liver cirrhosis. Intraabdominal hypertension and structural alterations of parenchyma involve decisive changes in hepatosplanchnic blood flow. Clearance of indo-cyanine green (ICG) is mainly dependent on hepatic perfusion and hepatocellular function. As a consequence, plasma disappearance rate of ICG (ICG-PDR) is rated as a useful dynamic parameter of liver function. This study primarily evaluates the impact of large-volume paracentesis (LVP) on ICG-PDR in critically ill patients with decompensated cirrhosis. Additionally, it describes influences on intraabdominal pressure (IAP), abdominal perfusion pressure (APP), hepatic blood flow, hemodynamic and respiratory function.

**Methods:**

We analyzed LVP in 22 patients with decompensated liver cirrhosis. ICG-PDR was assessed by using noninvasive LiMON technology (Pulsion^®^ Medical Systems; Maquet Getinge Group), and hepatic blood flow was analyzed by color-coded duplex sonography.

**Results:**

Paracentesis of a median volume of 3450 mL ascites evoked significant increases of ICG-PDR from 3.6 (2.8–4.6) to 5.1 (3.9–6.2)%/min (*p* < 0.001). Concomitantly, we observed a raise in “ICG-Clearance” from 99 (73.5–124.5) to 104 (91–143.5) mL/min/m^2^ (*p* = 0.005), while circulating blood volume index was unchanged [2412 (1983–3025) before paracentesis vs. 2409 (1997–2805) mL/m^2^, *p* = 0.734]. Sonography revealed a significant impact of paracentesis on hepatic blood flow: Hepatic artery resistance index dropped from 0.74 (0.68–0.75) to 0.68 (0.65–0.71) (*p* < 0.001) and maximum flow velocity in hepatic vein increased from 24 (17–30) to 30 (22–36) cm/s (*p* < 0.001). Consistent with previous studies, paracentesis caused significant decreases in IAP from 19.0 (15.0–20.3) to 11.0 (8.8–12.3) mmHg (*p* < 0.001) and central venous pressure from 22.5 (17.8–29.0) to 17.5 (12.8–24.0) mmHg (*p* < 0.001) with inverse increases in APP from 63.0 (56.8–69.5) to 71.0 (65.5–78.5) mmHg (*p* < 0.001). Changes in ICG-PDR were concomitant with changes in IAP (*r* = − 0.602) and APP (*r* = 0.576). Moreover, we found a substantial improvement in respiratory function. By contrast, hemodynamic parameters assessed by transpulmonary thermodilution, serum bilirubin and international normalized ratio did not change after paracentesis.

**Conclusion:**

Critically ill patients with decompensated cirrhosis and elevated IAP showed dramatically impaired ICG-PDR. Paracentesis evoked an improvement in ICG-PDR in parallel with a decreased IAP and an increased APP, while conventional parameters of liver function did not change. This effect on ICG-PDR is mainly referable to a relief of intraabdominal hypertension and changes in hepatosplanchnic blood flow.

## Background

Decompensated liver cirrhosis implies serious consequences for affected patients. Ascites is one major and highly frequently emerging complication [1, 2]. Intraabdominal hypertension (IAH) is associated with poor prognosis and high mortality [3, 4]. Increased intraabdominal pressure (IAP) involves multiple organ dysfunction regarding cardiovascular, respiratory, renal and abdominal impairment [5–8]. In particular, IAH interferes with proper abdominal perfusion, including hepatosplanchnic blood flow [9, 10].

Furthermore, advanced cirrhosis is accompanied by structural alterations leading to increased intrahepatic vascular resistance [11, 12]. Restrictions of intrahepatic blood flow cause portal hypertension, further aggravated by compensatory splanchnic arterial vasodilation [13]. Moreover, cirrhosis provokes impairment of blood flow in hepatic veins as well as increases in hepatic artery resistance index [14, 15]. Color-coded duplex sonography provides a subjective, but noninvasive diagnostic approach regarding vascular disorders in advanced liver disease [16].

Finally, end-stage liver disease is associated with progressive loss of functional liver capacity. Conventional laboratory assessment of liver function is mainly based on liver enzymes, bilirubin and the coagulation parameter international normalized ratio (INR) [17]. Dynamic tests of liver function at bedside might be more precise and objective relating to actual, short-term functional status [18]. Promising experiences were achieved with noninvasive measurement of plasma disappearance rate of indo-cyanine green (ICG-PDR). ICG is injected intravenously, distributed via blood circulation and excreted hepatobiliary [19]. ICG-PDR reflects both hepatosplanchnic blood flow and hepatocellular and excretory function [20, 21]. Some previous data described an interaction of ICG-PDR with intraabdominal pressure level [22–24]. Recently, one of these studies affirmed that ICG-PDR correlates inversely with IAP, suggesting that IAH restrains hepatosplanchnic and sinusoidal perfusion [25]. The abdominal perfusion pressure (APP), defined as difference between mean arterial pressure (MAP) and IAP, was positively correlated with ICG-PDR.

The accumulation of ascites is a typical complication of decompensated cirrhosis with elevated IAP and restricted organ perfusion [2, 10]. The evacuation of ascites by large-volume paracentesis (LVP) is one of the few nonsurgical treatment options [26]. ICG-PDR was already labeled as an accurate test for prediction of survival in advanced cirrhosis [27]. So far, none of the previous studies focused on the impact of LVP on dynamic liver assessment by ICG-PDR in this patient population. Consequently, the aim of our study was to investigate the effect of LVP on ICG-PDR in critically ill patients with decompensated liver cirrhosis (primary endpoint). This evaluation was supplemented by analyses of IAP, APP, hepatic blood flow by sonography, respiratory function as well as hemodynamic monitoring by using transpulmonary thermodilution.

## Methods

### Study design

This observational study was approved by the institutional review board (Ethikkommission Technische Universität München; Fakultät für Medizin; Project Number 5384/12), and informed consent was obtained by all patients.

Between April 2016 and July 2017, a total of 29 critically ill patients with decompensated liver cirrhosis on our university hospital general ICU were screened for the feasibility of LVP, analyses of ICG-PDR, IAP and hemodynamic monitoring via transpulmonary thermodilution. LVP was performed irrespective of the study based on the indication made by the treating ICU physician. We released a maximum of mobilizable ascites in each individual case of LVP; laboratory analyses of ascites revealed a cell count < 500/μL and polymorphonuclear neutrophils < 250/μL in every single patient. Due to potential influences on hepatosplanchnic blood flow, analysis was considered feasible only in patients without terlipressin treatment, portal vein thrombosis or transjugular intrahepatic portosystemic stent shunt. Therefore, 6 patients with terlipressin-treatment, 1 patient with portal vein thrombosis and 2 patients with portosystemic stent were restrained from the study. Finally, we analyzed a total of 22 critically ill patients with decompensated liver cirrhosis and tense ascites.

### Techniques

#### Assessment of ICG-PDR, BVI, CBI and laboratory tests

ICG-PDR, circulating blood volume index (BVI) and “ICG-Clearance” (CBI) were analyzed immediately before and after paracentesis by using noninvasive LiMON technology (Pulsion^®^ Medical Systems; Maquet Getinge Group) via a disposable finger color sensor as previously described [28]. We used ICG solubilized in distilled water and injected via a central venous catheter at a dose of 0.5 mg/kg of ICG-solution for measurement of ICG-PDR and additional assessment of BVI and CBI. Normally, ICG-PDR amounts to 18–25%/min [20]. Measurements of BVI and CBI were performed in parallel with ICG-PDR after manual input of current cardiac output and automatically indexed according to manufacturer’s recommendations.

Main laboratory tests of excretory liver function and synthesis (Bilirubin, INR) were analyzed once a day—corresponding to current standard in our intensive care unit. Overall time interval between blood sampling was 24 h.

#### LVP, IAP, abdominal compliance, APP and CVP

LVP was performed ultrasound guided after bringing the patient in supine position [29]. IAP was determined by intravesical measurement using a home-made technique according to The Abdominal Compartment Society (WSACS), and IAH was defined as IAP ≥ 12 mmHg [30, 31]. Abdominal compliance was expressed as the change of ascites volume per change in IAP (delta IAP) before and after paracentesis [32]. APP was calculated as MAP minus IAP by using concomitantly obtained values as explained previously [25, 31]. Substitution of albumin followed current guidelines and was performed after the final analyses [33]. Central venous pressure (CVP) was measured via jugular central venous catheters at end-expiration.

#### Ventilator setting and respiratory function

Patients with spontaneous breathing received a demand-based application of oxygen. Mechanical ventilation was performed using the routine ventilator device EVITA XL of our ICU (Dräger, Lübeck, Germany). Parameters were set according to current ARDSNet recommendations, especially regarding positive end-expiratory pressure (PEEP) [34]. Ventilator setting was based on medical assessment by the treating ICU physician irrespective of the study. The EVITA XL ventilator continuously monitored levels of airway pressures and corresponding volumes. Routine ventilatory parameters such as PEEP, tidal volume (TV), mean airway pressure, dynamic respiratory system compliance (C_dyn_) and fraction of inspired oxygen (F_i_O_2_) were recorded at baseline and at the end of LVP. P_a_O_2_ and P_a_CO_2_ were derived from a fully automatic blood gas analysis device (Rapid Point 400, Siemens Healthcare Diagnostic GmbH, Eschborn, Germany). Blood gas analysis and ventilatory parameters were used for calculation of Horowitz-index (P_a_O_2_/F_i_O_2_) and oxygenation index (OI = F_i_O_2_ * mean airway pressure * 100/P_a_O_2_) [35].

#### Hemodynamic monitoring

With the exception of only a single subject, all patients were under hemodynamic monitoring irrespective of the study, by using transpulmonary thermodilution with the PiCCO-2-device (Pulsion Medical Systems SE, Maquet Getinge Group) as described previously [36]: A 5 Fr thermistor-tipped arterial line (Pulsiocath, Pulsion^®^ Medical Systems; Maquet Getinge Group) inserted through a femoral artery and a hemodynamic monitor (PiCCO-2, Pulsion^®^ Medical Systems, Maquet Getinge Group) served to derive and analyze the thermodilution curve after injection of a cold indicator bolus (15 ml of saline cooled down to 4 °C) through a jugular central venous catheter. Measurements were done in triplicate, averaged and automatically indexed according to manufacturer’s recommendations.

#### Color-coded duplex sonography

Transabdominal ultrasound examination was accomplished noninvasively at the bedside in a supine position of the patients. All analyses were performed by a single physician with 6 years of institutional experience in the field of abdominal ultrasound. We used the mobile ultrasound scanner ACUSON X300 (Siemens Healthcare GmbH, Erlangen, Germany) and a convex 3.5 MHz transducer with color Doppler capacity. The transducer was placed in the right intercostal space due to the intraabdominal fluid accumulation. Doppler-analyses of blood flow were performed of the portal vein, hepatic artery and right hepatic vein; middle hepatic vein was chosen only when analysis of right hepatic vein was insufficient [14].

#### Data collection

Clinical and laboratory parameters for the calculation of APACHE II-, SOFA-, MELD- and Child–Pugh scores were recorded on the day of paracentesis. Measurements of ICG-PDR, BVI and CBI were done immediately before and after LVP, with a median time interval of 210 (180–255) min. Ventilatory parameters, hemodynamic profiles as well as IAP-assessment and ultrasound examinations were performed immediately before as well as after the maximal mobilizable release of ascites.

### Statistical analysis and primary endpoint

For primary outcome analysis, we investigated ICG-PDR at the end of LVP compared to baseline. All analyses and graphs were generated using GraphPad Prism 7.0 (GraphPad Software, La Jolla, CA, USA). Correlations were calculated using Pearson`s correlation coefficient *r* and linear regressions using the coefficient *R*^2^. Continuous variables are expressed as median and interquartile range (IQR). Categorical variables are expressed as percentages. To compare continuous variables, we used nonparametric Wilcoxon test for paired samples. Significance was assumed at a *p* value < 0.05.

## Results

### Patients’ baseline characteristics

Patients’ baseline characteristics and clinical scores are presented in Table 1.

**Table 1:** Patients baseline characteristics and clinical scores

Patients characteristics	
Male sex [*n*/total (%)]	15/22 (68%)
Age (years)	55 (52–69.3)
Body weight (kg)	82.5 (70–100)
Body height (cm)	180 (170–183)
APACHE II	24 (19–29.3)
SOFA	12 (9.8–16)
MELD	27.5 (23.8–36.3)
Child–Pugh	12 (10–13)
Child C [*n*/total (%)]	19/22 (86%)
Etiology of cirrhosis [*n*/total (%)]	Alcoholic 16/22 (72%)
	Viral 3/22 (14%)
	Cryptogenic 3/22 (14%)
Admission diagnoses [*n*/total (%)]	Sepsis/Pneumonia 10/22 (45%)
	Acute kidney failure 7/22 (32%)
	Hepatic encephalopathy 5/22 (23%)
Mode of ventilation [*n*/total (%)]	Spontaneous breathing 6/22 (27%)
	Pressure-supported 6/22 (27%)
	Pressure-controlled 10/22 (46%)
Ascites volume (mL)	3450 (3075–4700)
Total cell count (*n*/μL)	180 (100–310)
PEEP-level (cmH_2_O)	8 (8–10)
Baseline F_i_O_2_ (%)	45 (35–60)

*APACHE* acute physiology and chronic health evaluation, *SOFA* sequential organ failure assessment, *MELD* model of end-stage liver disease, *PEEP* positive end-expiratory pressure, *F*_*i*_*O*_*2*_ fraction of inspired oxygen

We performed LVP procedures in a total of 22 patients (7 female, 15 male) with decompensated liver cirrhosis and tense ascites. APACHE-, SOFA-, MELD- and Child–Pugh scores are explainable by advanced hepatic impairment and critical illness. The etiology of cirrhosis was predominantly alcoholic-toxic. About 75% of patients were mechanically ventilated and about 25% were spontaneously breathing. Regarding mechanically ventilated patients, PEEP-setting was unchanged during LVP and study measurements.

### LVP, ICG-PDR, BVI, CBI and laboratory tests

Twenty-two LVP procedures with a median volume of 3450 (3075–4700) mL removed ascites (≥ 3000 mL for every paracentesis) were analyzed. We noticed a median ascitic cell count of 180 (100–310)/μL (≤ 500**/**μL in all patients), with polymorphonuclear neutrophils < 250/μL in each patient to exclude spontaneous bacterial peritonitis and to justify a release of a maximum of mobilizable ascites volume.

**Fig. 1:**
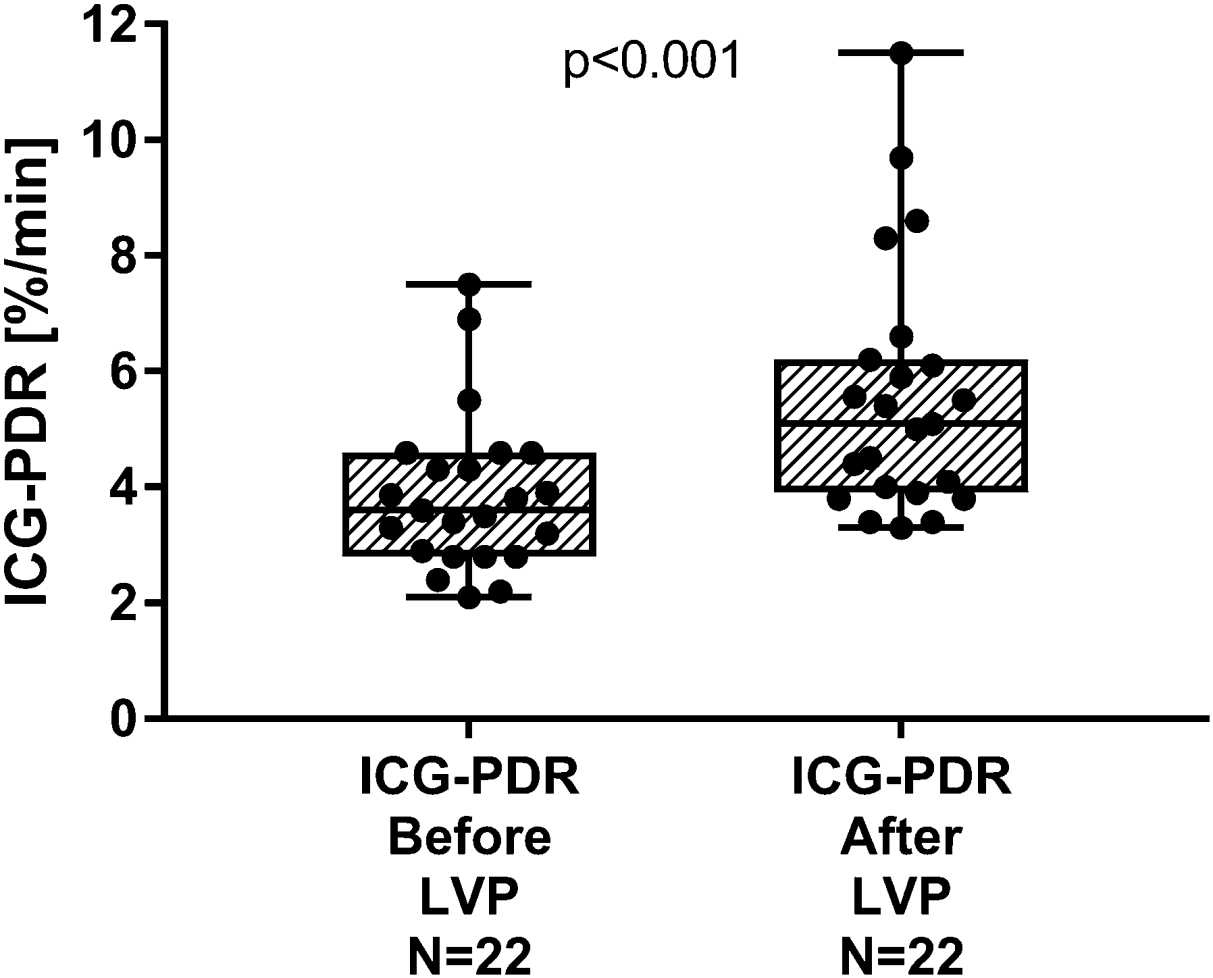
ICG-PDR at baseline and after large-volume paracentesis (LVP), depicted as box plots (median and IQR, min to max) and showing all individual points

ICG-PDR at baseline was decreased substantially to 3.6 (2.8–4.6)%/min compatible with dramatic hepatic impairment of advanced liver cirrhosis. LVP provoked a significant increase of ICG-PDR to 5.1 (3.9–6.2)%/min (*p* < 0.001) (Fig. 1). Median change of ICG-PDR (delta ICG-PDR) induced by LVP was 1.2 (1.0–2.1)%/min.

**Fig. 2:**
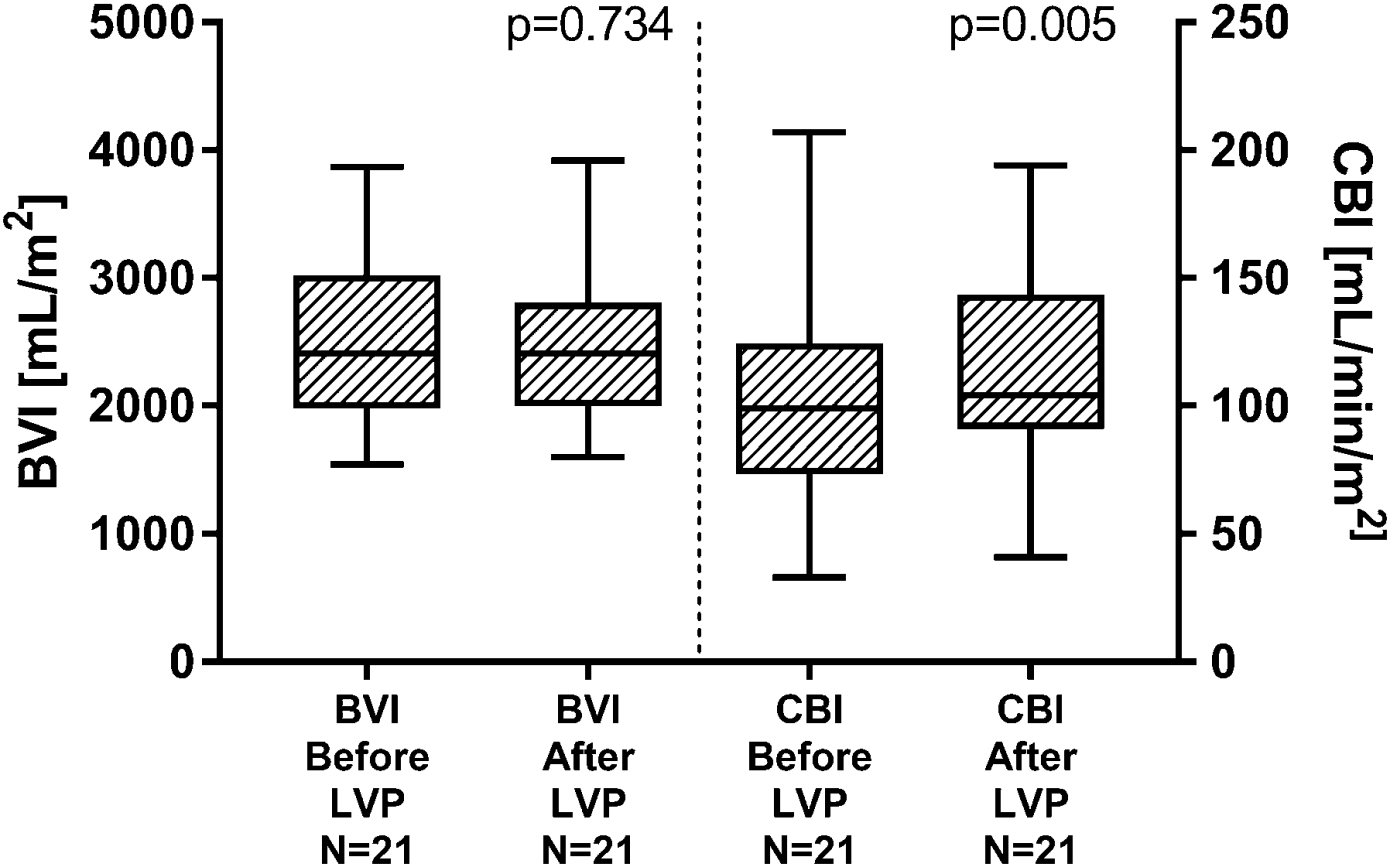
Circulating blood volume index (BVI) and “ICG-Clearance” (CBI) before and after large-volume paracentesis (LVP), depicted as box plots (median and IQR, min to max)

Circulating blood volume index (BVI) was unchanged after LVP [2412 (1983–3025) before paracentesis vs. 2409 (1997–2805) mL/m^2^, *p* = 0.734], while “ICG-Clearance” (CBI) increased from 99 (73.5–124.5) to 104 (91–143.5) mL/min/m^2^ (*p* = 0.005) (Fig. 2).

In comparison, main laboratory tests of liver function showed no significant changes after an overall time interval of 24 h: Parameter of liver excretion function bilirubin as well as plasmatic coagulation parameter INR remained stable (Table 2).

**Table 2:** Main conventional laboratory parameters of liver function before and after paracentesis (overall time interval of 24 h)

Conventional laboratory tests of hepatic function			
	Before paracentesis	After paracentesis	*p* value
	Median (IQR)	Median (IQR)	
Bilirubin (mg/dL)	7.8 (3.3–19.0)	7.6 (2.5–21.4)	0.868
INR	1.8 (1.4–2.2)	1.8 (1.4–2.5)	0.094

*INR* international normalized ratio

### IAP, abdominal compliance, APP and CVP

Paracentesis caused a distinct relief of IAH: At the end of LVP, pressure levels of IAP had lowered from 19.0 (15.0–20.3) to 11.0 (8.8–12.3) mmHg (*p* < 0.001). Grading of IAH before and after LVP is listed in Table 3, according to the definition established by the WSACS [31]. Median abdominal compliance based on paracentesis of 3450 (3075–4700) mL was 461 (383–659) mL/mmHg.

**Table 3:** Grading of intraabdominal hypertension before and after paracentesis

Grading of IAH according to WSACS definition		
	Before LVP	After LVP
	*n*/total (%)	*n*/total (%)
No IAH, IAP ≤ 11 mmHg	1/22 (5%)	13/22 (59%)
IAH Grade I, IAP 12–15 mmHg	5/22 (23%)	6/22 (27%)
IAH Grade II, IAP 16–20 mmHg	11/22 (50%)	2/22 (9%)
IAH Grade III, IAP 21–25 mmHg	3/22 (13%)	1/22 (5%)
IAH Grade IV, IAP > 25 mmHg	2/22 (9%)	0/22 (0%)

*IAH* intraabdominal hypertension, *WSACS* The Abdominal Compartment Society, *LVP* large-volume paracentesis, *IAP* Intraabdominal pressure

**Fig. 3:**
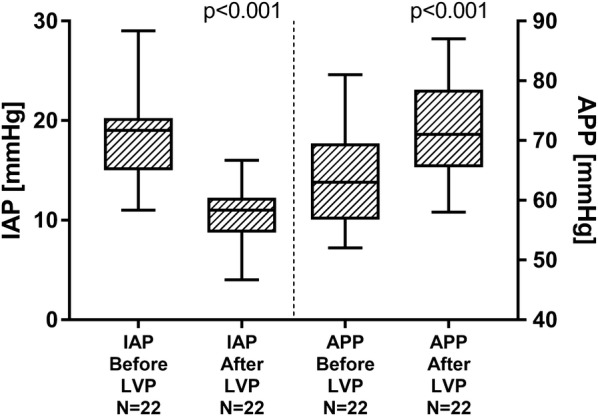
Intraabdominal pressure (IAP) and abdominal perfusion pressure (APP) before and after large-volume paracentesis (LVP), depicted as box plots (median and IQR, min to max)

Consecutively, we noticed a marked improvement of APP from 63.0 (56.8–69.5) to 71.0 (65.5–78.5) mmHg (*p* < 0.001). Pressure levels of IAP and APP are depicted in Fig. 3. In parallel with the decline of IAP, LVP induced a significant decrease of CVP from 22.5 (17.8–29.0) to 17.5 (12.8–24.0) mmHg (*p* = 0.001).

Median changes in IAP (delta IAP) and APP (delta APP) caused by paracentesis were -8.0 (− 5.0 to − 10.0) mmHg and 8.5 (5.8–10.3) mmHg, respectively. Furthermore, LVP provoked a median change in CVP (delta CVP) of − 5.0 (− 3.0 to − 7.0) mmHg.

### Correlations and regression plots

**Fig. 4:**
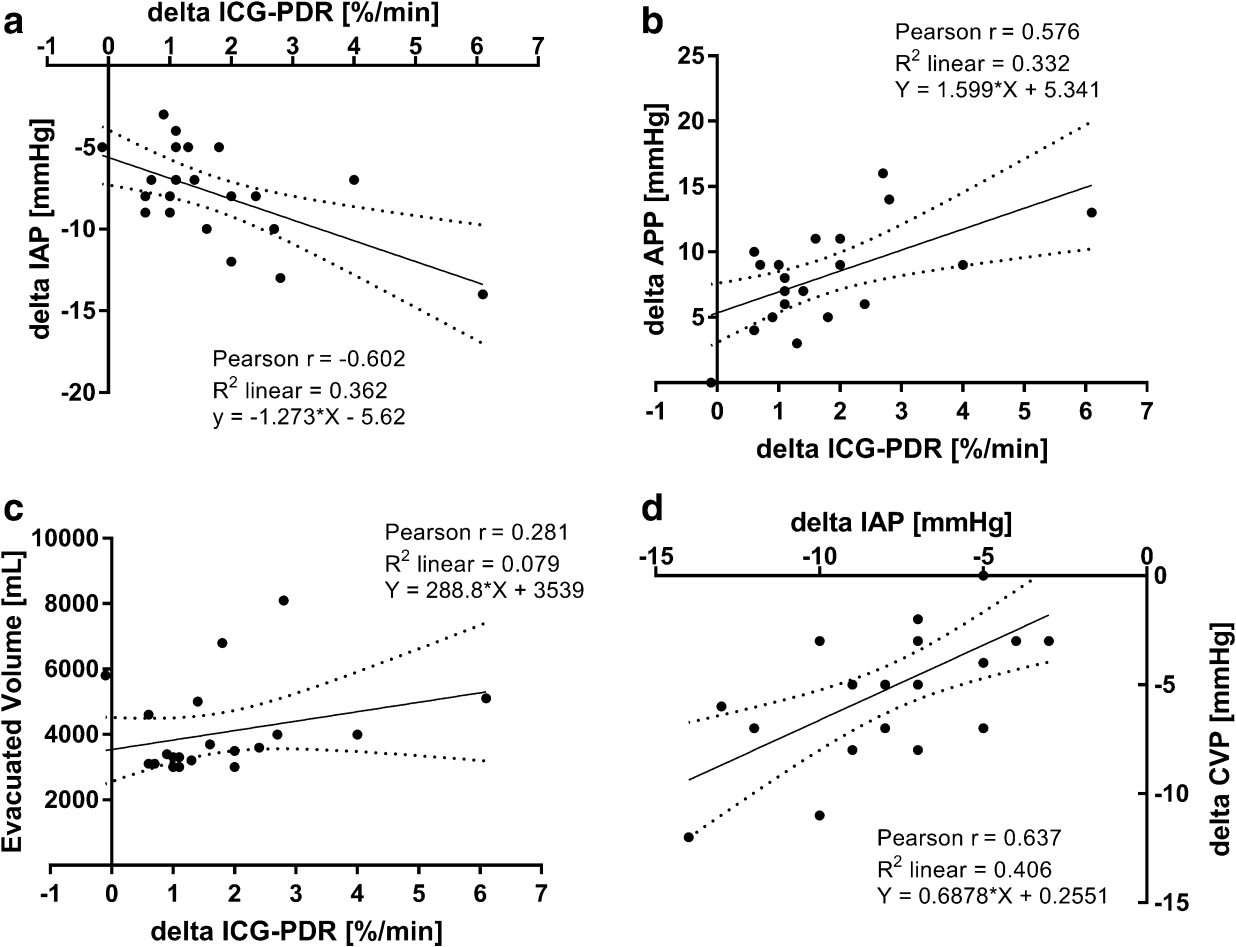
Pearson correlations and regression plots of changes caused by paracentesis per patient (*n* = 22). **a** delta ICG-PDR correlated with delta IAP. **b** delta ICG-PDR correlated with delta APP. **c** delta ICG-PDR correlated with evacuated volume. **d** delta IAP correlated with delta CVP

Analyses according to Pearson as well as linear regressions are illustrated in Fig. 4. Paracentesis of a median volume of 3450 (3075–4700) mL in a total of 22 patients provoked concomitant changes of ICG-PDR (delta ICG-PDR) with changes in IAP (panel A, *r* = − 0.602, *p* = 0.003). In parallel, delta ICG-PDR correlated significantly with delta APP (panel B, *r* = 0.576, *p* = 0.005). In contrast, delta ICG-PDR was not significantly associated with evacuated ascites volume (panel C, *r* = 0.281, *p* = 0.205). Concerning concomitant changes of IAP and CVP after LVP, correlation analyses outlined an association of delta IAP and delta CVP (panel D, *r* = 0.637, *p* = 0.001).

### Color-coded duplex sonography

By sonographic examination, we registered a significant impact of LVP on hepatic blood flow: Hepatic artery resistance index dropped from 0.74 (0.68–0.75) to 0.68 (0.65–0.71) (*p* < 0.001). This reduction was mainly reflected in an increase of diastolic hepatic arterial flow velocity, while systolic arterial flow velocity was steady. Furthermore, LVP provoked an increase of maximum hepatic vein flow velocity. In contrast, maximum flow velocity in portal vein was mainly unaffected by paracentesis (Table 4).

**Table 4:** Ultrasound examination of hepatic blood flow by color-coded duplex sonography of hepatic artery, portal vein and hepatic vein before and after paracentesis

Color-coded duplex sonography of hepatic blood flow			
	Before paracentesis	After paracentesis	*p* value
	Median (IQR)	Median (IQR)	
HARI	0.74 (0.68–0.75)	0.68 (0.65–0.71)	< 0.001
Systolic HAF (cm/s)	129 (115–145)	123 (114–140)	0.100
Diastolic HAF (cm/s)	35 (24–48)	40 (31–50)	0.009
PVF (cm/s)	20 (16–28)	21 (15–32)	0.753
HVF (cm/s)	24 (17–30)	30 (22–36)	< 0.001

*HARI* hepatic artery resistance index, *HAF* maximum hepatic arterial flow velocity, *PVF* maximum portal vein flow velocity, HVF: Maximum hepatic vein flow velocity

### Respiratory and ventilatory parameters

Respiratory function improved by paracentesis without changes of PEEP-level, outlined in Table 5. Horowitz-index (P_a_O_2_/F_i_O_2_) increased and oxygenation index (OI) improved. Furthermore, we registered a significant raise in TV and C_dyn_. We also recorded a decrease in respiratory rate and P_a_CO_2_, but results were not statistically significant.

**Table 5:** Respiratory and ventilatory parameters before and after paracentesis

Respiratory and ventilatory parameters			
	Before paracentesis	After paracentesis	*p* value
	Median (IQR)	Median (IQR)	
P_a_O_2_/F_i_O_2_	220 (126–271)	247 (138–321)	< 0.001
OI (cmH_2_O/mmHg)	8.0 (4.8–12.3)	5.8 (3.8–11.1)	< 0.001
TV (mL)	491 (337–542)	530 (414–590)	0.001
C_dyn_ (mL/cmH_2_O)	41 (21–46)	49 (24–65)	< 0.001
Respiratory rate (min^−1^)	24 (18–26)	22 (16–26)	0.062
P_a_CO_2_	36.7 (32.6–46.4)	37.0 (32.7–41.2)	0.115

*OI* oxygenation index, *TV* tidal volume, *C*_*dyn*_ dynamic respiratory system compliance, *P*_*a*_*CO*_*2*_ arterial partial pressure of carbon dioxide

### Hemodynamic parameters

Hemodynamic assessment by transpulmonary thermodilution and pulse contour analysis revealed overall unchanged parameters of hemodynamic function after LVP: Mean arterial pressure MAP, cardiac Index CI, cardiac output CO, global end-diastolic volume index GEDVI, extravascular lung water index EVLWI and systemic vascular resistance index SVRI did not change significantly (Table 6).

**Table 6:** Parameters of hemodynamic monitoring before and after paracentesis

Hemodynamic parameters assessed by transpulmonary thermodilution			
	Before paracentesis	After paracentesis	*p* value
	Median (IQR)	Median (IQR)	
MAP (mmHg)	82 (76–91)	79 (74–91)	0.134
CI (L/min/m^2^)	4.5 (3.8–6.5)	5.0 (3.8–6.6)	0.522
CO (L/min)	8.9 (7.5–13.1)	10.3 (7.3–13.4)	0.579
GEDVI (mL/m^2^)	880 (788–1021)	902 (758–1021)	0.437
EVLWI (mL/kg)	12 (9.5–15)	12 (9.5–14.5)	0.918
SVRI (dyn * s * cm^−5^ * m^−2^)	978 (725–1254)	1064 (713–1236)	0.772
CVP (mmHg)	22.5 (17.8–29.0)	17.5 (12.8–24)	0.001

*CI* cardiac index, *CO* cardiac output, *GEDVI* global end-diastolic volume index, *EVLWI* extravascular lung water index, *SVRI* systemic vascular resistance index, *CVP* central venous pressure

## Discussion

The present study shows that large-volume paracentesis (LVP) induced an improvement in ICG-PDR in critically ill patients with decompensated liver cirrhosis. This effect comes along with a relief of intraabdominal hypertension (IAH) reflected in a decrease in intraabdominal pressure (IAP) and an inverse raise of abdominal perfusion pressure (APP).

ICG-PDR represents a useful dynamic liver test in addition to conventional laboratory parameters [17]. On the one hand, ICG-PDR is dependent on hepatocellular function [18–20, 37, 38]. On the other hand, it is highly influenced by sufficient hepatosplanchnic blood flow and sinusoidal perfusion [18, 21, 39]. Normal range of ICG-PDR is between 18 and 25%/min. Advanced liver cirrhosis involves severe decreases of ICG-PDR with consecutively grave consequences on patients outcome [20].

This dramatic impairment of ICG-PDR in case of end-stage liver disease is confirmed in our study. Median baseline ICG-PDR was reduced markedly to 3.6%/min. Large-volume paracentesis (LVP) provoked a significant increase in ICG-PDR. Additionally, we noticed an increase in “ICG-Clearance” in contrast to unchanged circulating blood volume index, but data on the relevance of these parameters are rare so far. As opposed to this, paracentesis had no influence on main laboratory parameters of hepatic function. In this context, it should be pointed out that an earlier study found no correlation between ICG-PDR and standard laboratory liver tests [25].

The improvement in ICG-PDR is in parallel with a decline of IAP after evacuation of ascites. Correlation analyses revealed a statistically significant association of changes in ICG-PDR with changes in IAP. Previously, a few studies described a relationship between ICG-PDR and IAP. Some of them focused on the effect of prone positioning on IAP and ICG-PDR in patients with respiratory failure [22, 40, 41]. Two studies characterized the inverse correlation of ICG-PDR with the dimension of IAP in critically ill patients [24, 25]. Sakka presented a case of abdominal compartment syndrome with impaired ICG-PDR and a significant increase after surgical relief [42]. Not less interesting is another report from the same author describing the beneficial effect of paracentesis on ICG-PDR in a woman with ascites due to chronic heart failure; while IAP dropped from 18 to 12 mmHg, ICG-PDR rose from 11.6 to 15.6%/min [23]. The basic rationale behind all these findings seems to be that IAH compromises APP and consecutively restrains hepatosplanchnic blood flow and sinusoidal perfusion [25]. According to this, decompression of abdomen with a decrease in IAP improves hepatic blood flow and increases ICG-PDR [23].

The results of our study reaffirmed previous trials concerning a significant and immediate drop of IAP after LVP in patients with decompensated cirrhosis and tense ascites [27, 30, 43]. The relief of IAH is paralleled by a significant raise of APP, suggesting a substantial effect on hepatic perfusion. In the present study, we used color-coded duplex sonography for analyses of liver blood flow. At baseline, we found an elevated hepatic artery resistance index. This observation is in line with former ultrasound examinations showing increased resistance index in case of advanced liver fibrosis and cirrhosis [15, 44–46]. The alterations of intrahepatic circulation in cirrhosis with endothelial dysfunction and increased vasoconstrictor activity are well established [13, 47]. We recognized a significant decrease in hepatic artery resistance index after LVP, mainly referable to a change in diastolic blood flow. The hypothesis behind this finding is that LVP reduces hepatic vascular resistance and enhances sinusoidal perfusion. Moreover, sonography revealed a raise in maximum blood flow velocity in hepatic vein after LVP. Damping of hepatic venous waveform is a frequent observation in advanced cirrhosis, but the significance of blood flow velocity in hepatic vein alone is not investigated so far [14, 48]. In contrast, there was no relevant change of portal vein flow after paracentesis, indicating an obvious portal hypertension in the studied population with advanced liver cirrhosis.

Considered as secondary endpoints of this study, we analyzed the impact of LVP on respiratory and circulatory function in case of decompensated cirrhosis. We registered an overall beneficial effect on parameters of oxygenation and ventilation. Several studies underlined that respiratory improvement was particularly attributable to decreases in IAP, enhanced ventilatory mechanics with increased compliance as well as alveolar recruitment with increased end-expiratory lung volume [43, 49–51]. Concerning hemodynamic function, previous studies yielded diverging results about the existing threat of paracentesis-induced cardiocirculatory dysfunction [52–54]. Nevertheless, a recent study demonstrated that LVP did not impair hemodynamic parameters assessed by transpulmonary thermodilution [43]. Our analyses via transpulmonary thermodilution reconfirmed this favorable situation with steady parameters of hemodynamic function after LVP. However, we noticed a significant decrease in central venous pressure after LVP, most probably due to the decrease in IAP and therefore extra-thoracic pressure level. In line with this, we found a significant association of changes in IAP with changes in central venous pressure.

Altogether, the present study emphasizes the inverse correlation of ICG-PDR with IAP and the far-reaching effects of IAH in critically ill patients with decompensated cirrhosis. Hydropic decompensation evokes harmful increases of IAP with negative effects on abdominal perfusion and liver blood flow. LVP immediately lowers IAP in combination with an increase in ICG-PDR. According to ultrasound examination, this beneficial effect on ICG-PDR is mainly referable to improved arterial liver perfusion and decreased hepatic vascular resistance. By implication, our study to some extent questions the significance of ICG-PDR for an exclusive evaluation of hepatocellular function in case of IAH.

### Strengths and limitations

To our knowledge, this is the first study evaluating the effects of LVP on ICG-PDR in a characterized population of critically ill patients with decompensated cirrhosis and tense ascites. The study combines ICG-PDR with assessments of IAP and APP, ultrasound examinations as well as respiratory and advanced hemodynamic monitoring. Despite the overall modest beneficial effect of paracentesis on ICG-PDR, the results are conclusive with high levels of statistical significance.

However, this is a single-center study with consecutively a very limited number of patients. Paracentesis was performed with a maximum release of mobilizable ascites instead of a stepwise release of predefined fluid amounts. Therefore, our data allowed only the calculation of an “overall” abdominal compliance with limited validity considering its evolution during progressive, stepwise evacuation of ascites [32]. Moreover, this study provides no further information about a possible influence of LVP on kidney function. In light of highly frequently occurring hepatorenal syndrome in case of decompensated liver cirrhosis, further studies would be interesting to investigate the impact of paracentesis on renal perfusion. In consideration of a relatively high baseline EVLWI in our patients, hemodynamic monitoring via transpulmonary thermodilution would have been even more accurate when performed with a higher saline bolus [55]. Ultrasound examinations via color-coded duplex sonography are operator-dependent and were performed only by one physician. Beside ICG-PDR, reliable data on additional parameters CBI and BVI provided by LiMON technology are rare.

## Conclusion

Decompensated cirrhosis is associated with a marked decrease of dynamic liver test ICG-PDR, reflecting reduced hepatocellular capacity as well as impaired hepatosplanchnic blood flow. LVP evokes a modest but significant improvement in ICG-PDR, primarily referable to a decline in IAP and an inverse increase in APP. While conventional laboratory parameters of liver function did not change, the increase in ICG-PDR is mainly attributable to changes in hepatic perfusion.

Moreover, LVP induced substantial improvement in respiratory parameters, while hemodynamic profiles remained stable.

## Abbreviations

IAH: intraabdominal hypertension
IAP: intraabdominal pressure
INR: international normalized ratio
ICG: indo-cyanine green
PDR: plasma disappearance rate
APP: abdominal perfusion pressure
MAP: mean arterial pressure
LVP: large-volume paracentesis
ICU: intensive care unit
BVI: circulating blood volume index
CBI: “ICG-Clearance”
WSACS: The Abdominal Compartment Society
CVP: central venous pressure
ARDS: acute respiratory distress syndrome
PEEP: positive end-expiratory pressure
TV: tidal volume
C_dyn_: dynamic respiratory system compliance
F_i_O_2_: fraction of inspired oxygen
P_a_O_2_: arterial partial pressure of oxygen
P_a_CO_2_: arterial partial pressure of carbon dioxide
OI: oxygenation Index
APACHE: acute and physiology chronic health evaluation
SOFA: sequential organ failure assessment
MELD: model of end-stage liver disease
IQR: interquartile range
HARI: hepatic artery resistance index
HAF: maximum hepatic arterial flow
PVF: maximum portal vein flow
HVF: maximum hepatic vein flow
CI: cardiac index
CO: cardiac output
GEDVI: global end-diastolic volume index
EVLWI: extravascular lung water index
SVRI: systemic vascular resistance index

## References

[1] Gines P, Quintero E, Arroyo V, Teres J, Bruguera M, Rimola A (1987). Compensated cirrhosis: natural history and prognostic factors. Hepatology.

[2] Bernardi M, Moreau R, Angeli P, Schnabl B, Arroyo V (2015). Mechanisms of decompensation and organ failure in cirrhosis: from peripheral arterial vasodilation to systemic inflammation hypothesis. J Hepatol.

[3] Malbrain ML, Chiumello D, Pelosi P, Bihari D, Innes R, Ranieri VM (2005). Incidence and prognosis of intraabdominal hypertension in a mixed population of critically ill patients: a multiple-center epidemiological study. Crit Care Med.

[4] Malbrain ML, Chiumello D, Pelosi P, Wilmer A, Brienza N, Malcangi V (2004). Prevalence of intra-abdominal hypertension in critically ill patients: a multicentre epidemiological study. Intensive Care Med.

[5] Rouby JJ, Constantin JM, De Roberto AGC, Zhang M, Lu Q (2004). Mechanical ventilation in patients with acute respiratory distress syndrome. Anesthesiology.

[6] Barnes GE, Laine GA, Giam PY, Smith EE, Granger HJ (1985). Cardiovascular responses to elevation of intra-abdominal hydrostatic pressure. Am J Physiol.

[7] Malbrain ML, Cheatham ML, Kirkpatrick A, Sugrue M, Parr M, De Waele J (2006). Results from the international conference of experts on intra-abdominal hypertension and abdominal compartment syndrome. I. Definitions. Intensive Care Med.

[8] von Delius S, Karagianni A, Henke J, Preissel A, Meining A, Frimberger E (2007). Changes in intra-abdominal pressure, hemodynamics, and peak inspiratory pressure during gastroscopy in a porcine model. Endoscopy.

[9] Malbrain ML, Deeren D, De Potter TJ (2005). Intra-abdominal hypertension in the critically ill: it is time to pay attention. Curr Opin Crit Care.

[10] Cresswell AB, Wendon JA (2007). Hepatic function and non-invasive hepatosplanchnic monitoring in patients with abdominal hypertension. Acta Clin Belg.

[11] Bosch J (2007). Vascular deterioration in cirrhosis: the big picture. J Clin Gastroenterol.

[12] Iwakiri Y, Groszmann RJ (2006). The hyperdynamic circulation of chronic liver diseases: from the patient to the molecule. Hepatology.

[13] Iwakiri Y (2014). Pathophysiology of portal hypertension. Clin Liver Dis.

[14] Scheinfeld MH, Bilali A, Koenigsberg M (2009). Understanding the spectral Doppler waveform of the hepatic veins in health and disease. Radiographics.

[15] Zekanovic D, Ljubicic N, Boban M, Nikolic M, Delic-Brkljacic D, Gacina P (2010). Doppler ultrasound of hepatic and system hemodynamics in patients with alcoholic liver cirrhosis. Dig Dis Sci.

[16] Zwiebel WJ (1995). Sonographic diagnosis of hepatic vascular disorders. Semin Ultrasound CT MRI.

[17] Sakka SG (2007). Assessing liver function. Curr Opin Crit Care.

[18] Sakka SG, Klein M, Reinhart K, Meier-Hellmann A (2002). Prognostic value of extravascular lung water in critically ill patients. Chest.

[19] Stehr A, Ploner F, Traeger K, Theisen M, Zuelke C, Radermacher P (2005). Plasma disappearance of indocyanine green: a marker for excretory liver function?. Intensive Care Med.

[20] Faybik P, Hetz H (2006). Plasma disappearance rate of indocyanine green in liver dysfunction. Transplant Proc.

[21] Seibel A, Muller A, Sakka SG (2011). Indocyanine green plasma disappearance rate for monitoring hepatosplanchnic blood flow. Intensive Care Med.

[22] Michelet P, Roch A, Gainnier M, Sainty JM, Auffray JP, Papazian L (2005). Influence of support on intra-abdominal pressure, hepatic kinetics of indocyanine green and extravascular lung water during prone positioning in patients with ARDS: a randomized crossover study. Crit Care.

[23] Sakka SG (2006). Indocyanine green plasma disappearance rate during relief of increased abdominal pressure. Intensive Care Med.

[24] Inal MT, Memis D, Sezer YA, Atalay M, Karakoc A, Sut N (2011). Effects of intra-abdominal pressure on liver function assessed with the LiMON in critically ill patients. Can J Surg.

[25] Malbrain ML, Viaene D, Kortgen A, De Laet I, Dits H, Van Regenmortel N (2012). Relationship between intra-abdominal pressure and indocyanine green plasma disappearance rate: hepatic perfusion may be impaired in critically ill patients with intra-abdominal hypertension. Ann Intensive Care.

[26] Zipprich A, Kuss O, Rogowski S, Kleber G, Lotterer E, Seufferlein T (2010). Incorporating indocyanin green clearance into the Model for End Stage Liver Disease (MELD-ICG) improves prognostic accuracy in intermediate to advanced cirrhosis. Gut.

[27] Cheatham ML (2009). Nonoperative management of intraabdominal hypertension and abdominal compartment syndrome. World J Surg.

[28] Sakka SG, Reinhart K, Meier-Hellmann A (2000). Comparison of invasive and noninvasive measurements of indocyanine green plasma disappearance rate in critically ill patients with mechanical ventilation and stable hemodynamics. Intensive Care Med.

[29] Umgelter A, Reindl W, Wagner KS, Franzen M, Stock K, Schmid RM (2008). Effects of plasma expansion with albumin and paracentesis on haemodynamics and kidney function in critically ill cirrhotic patients with tense ascites and hepatorenal syndrome: a prospective uncontrolled trial. Crit Care.

[30] Malbrain ML (2004). Different techniques to measure intra-abdominal pressure (IAP): time for a critical re-appraisal. Intensive Care Med.

[31] Kirkpatrick AW, Roberts DJ, De Waele J, Jaeschke R, Malbrain ML, De Keulenaer B (2013). Intra-abdominal hypertension and the abdominal compartment syndrome: updated consensus definitions and clinical practice guidelines from the World Society of the Abdominal Compartment Syndrome. Intensive Care Med.

[32] Malbrain ML, Peeters Y, Wise R (2016). The neglected role of abdominal compliance in organ–organ interactions. Crit Care.

[33] Gines A, Fernandez-Esparrach G, Monescillo A, Vila C, Domenech E, Abecasis R (1996). Randomized trial comparing albumin, dextran 70, and polygeline in cirrhotic patients with ascites treated by paracentesis. Gastroenterology.

[34] Bein T, Grasso S, Moerer O, Quintel M, Guerin C, Deja M (2016). The standard of care of patients with ARDS: ventilatory settings and rescue therapies for refractory hypoxemia. Intensive Care Med.

[35] Bone RC, Maunder R, Slotman G, Silverman H, Hyers TM, Kerstein MD (1989). An early test of survival in patients with the adult respiratory distress syndrome. The P_a_O_2_/F_i_O_2_ ratio and its differential response to conventional therapy. Prostaglandin E1 Study Group. Chest.

[36] Huber W, Umgelter A, Reindl W, Franzen M, Schmidt C, von Delius S (2008). Volume assessment in patients with necrotizing pancreatitis: a comparison of intrathoracic blood volume index, central venous pressure, and hematocrit, and their correlation to cardiac index and extravascular lung water index. Crit Care Med.

[37] Hemming AW, Scudamore CH, Shackleton CR, Pudek M, Erb SR (1992). Indocyanine green clearance as a predictor of successful hepatic resection in cirrhotic patients. Am J Surg.

[38] Scheingraber S, Richter S, Igna D, Flesch S, Kopp B, Schilling MK (2008). Indocyanine green disappearance rate is the most useful marker for liver resection. Hepatogastroenterology.

[39] Inal MT, Memis D, Kargi M, Sut N (2009). Prognostic value of indocyanine green elimination assessed with LiMON in septic patients. J Crit Care.

[40] Hering R, Vorwerk R, Wrigge H, Zinserling J, Schroder S, von Spiegel T (2002). Prone positioning, systemic hemodynamics, hepatic indocyanine green kinetics, and gastric intramucosal energy balance in patients with acute lung injury. Intensive Care Med.

[41] Kirkpatrick AW, Pelosi P, De Waele JJ, Malbrain ML, Ball CG, Meade MO (2010). Clinical review: intra-abdominal hypertension: does it influence the physiology of prone ventilation?. Crit Care.

[42] Sakka SG (2007). Indocyanine green plasma disappearance rate as an indicator of hepato-splanchnic ischemia during abdominal compartment syndrome. Anesth Analg.

[43] Phillip V, Saugel B, Ernesti C, Hapfelmeier A, Schultheiss C, Thies P (2014). Effects of paracentesis on hemodynamic parameters and respiratory function in critically ill patients. BMC Gastroenterol.

[44] Sacerdoti D, Merkel C, Bolognesi M, Amodio P, Angeli P, Gatta A (1995). Hepatic arterial resistance in cirrhosis with and without portal vein thrombosis: relationships with portal hemodynamics. Gastroenterology.

[45] Lutz HH, Gassler N, Tischendorf FW, Trautwein C, Tischendorf JJ (2012). Doppler ultrasound of hepatic blood flow for noninvasive evaluation of liver fibrosis compared with liver biopsy and transient elastography. Dig Dis Sci.

[46] Glisic TM, Perisic MD, Dimitrijevic S, Jurisic V (2014). Doppler assessment of splanchnic arterial flow in patients with liver cirrhosis: correlation with ammonia plasma levels and MELD score. J Clin Ultrasound.

[47] Gracia-Sancho J, Lavina B, Rodriguez-Vilarrupla A, Garcia-Caldero H, Bosch J, Garcia-Pagan JC (2007). Enhanced vasoconstrictor prostanoid production by sinusoidal endothelial cells increases portal perfusion pressure in cirrhotic rat livers. J Hepatol.

[48] Kim MY, Baik SK, Park DH, Lim DW, Kim JW, Kim HS (2007). Damping index of Doppler hepatic vein waveform to assess the severity of portal hypertension and response to propranolol in liver cirrhosis: a prospective nonrandomized study. Liver Int.

[49] Levesque E, Hoti E, Jiabin J, Dellamonica J, Ichai P, Saliba F (2011). Respiratory impact of paracentesis in cirrhotic patients with acute lung injury. J Crit Care.

[50] Byrd RP, Roy TM, Simons M (1996). Improvement in oxygenation after large volume paracentesis. South Med J.

[51] Wauters J, Claus P, Brosens N, McLaughlin M, Hermans G, Malbrain M (2012). Relationship between abdominal pressure, pulmonary compliance, and cardiac preload in a porcine model. Crit Care Res Pract.

[52] Cabrera J, Falcon L, Gorriz E, Pardo MD, Granados R, Quinones A (2001). Abdominal decompression plays a major role in early postparacentesis haemodynamic changes in cirrhotic patients with tense ascites. Gut.

[53] Nasr G, Hassan A, Ahmed S, Serwah A (2010). Predictors of large volume paracantesis induced circulatory dysfunction in patients with massive hepatic ascites. J Cardiovasc Dis Res.

[54] Peltekian KM, Wong F, Liu PP, Logan AG, Sherman M, Blendis LM (1997). Cardiovascular, renal, and neurohumoral responses to single large-volume paracentesis in patients with cirrhosis and diuretic-resistant ascites. Am J Gastroenterol.

[55] Hofkens PJ, Verrijcken A, Merveille K, Neirynck S, Van Regenmortel N, De Laet I (2015). Common pitfalls and tips and tricks to get the most out of your transpulmonary thermodilution device: results of a survey and state-of-the-art review. Anaesthesiol Intensive Ther.

